# Replication and Adaptive Mutations of Low Pathogenic Avian Influenza Viruses in Tracheal Organ Cultures of Different Avian Species

**DOI:** 10.1371/journal.pone.0042260

**Published:** 2012-08-13

**Authors:** Henning Petersen, Mikhail Matrosovich, Stephan Pleschka, Silke Rautenschlein

**Affiliations:** 1 Clinic for Poultry, University of Veterinary Medicine Hannover, Hannover, Germany; 2 Institute of Virology, Philipps University Marburg, Marburg, Germany; 3 Institute for Medical Virology, Justus-Liebig-University Giessen, Giessen, Germany; The University of Hong Kong, China

## Abstract

Transmission of avian influenza viruses (AIV) between different avian species may require genome mutations that allow efficient virus replication in a new species and could increase virulence. To study the role of domestic poultry in the evolution of AIV we compared replication of low pathogenic (LP) AIV of subtypes H9N2, H7N7 and H6N8 in tracheal organ cultures (TOC) and primary embryo fibroblast cultures of chicken, turkey, Pekin duck and homing pigeon. Virus strain-dependent and avian species-related differences between LPAIV were observed in growth kinetics and induction of ciliostasis in TOC. In particular, our data demonstrate high susceptibility to LPAIV of turkey TOC contrasted with low susceptibility of homing pigeon TOC. Serial virus passages in the cells of heterologous host species resulted in adaptive mutations in the AIV genome, especially in the receptor-binding site and protease cleavage site of the hemagglutinin. Our data highlight differences in susceptibility of different birds to AIV viruses and emphasizes potential role of poultry in the emergence of new virus variants.

## Introduction

Wild aquatic birds are generally considered as the primary natural reservoir for avian influenza viruses (AIV) [1]. All known 16 hemagglutinin (HA) and 9 neuraminidase (NA) antigenic subtypes have been isolated from wild birds [1]–[4]. The majority of possible influenza virus HA- and NA-subtype combinations are found in birds of the order Anseriformes, most frequently in dabbling ducks such as the mallard (*Anas platyrhynchos*) [3], [4]. Interspecies transmission of AIV between wild bird populations and domestic poultry species is an occasional event [5]. Complex interactions between several virus and host factors are needed for successful AIV transmission and adaptation to new hosts [6]. Mutations in the virus genome as well as reassortment may allow viruses to cross species barriers, adapt to new hosts, and potentially increase virulence [7].

Susceptibility to influenza virus infection, development of clinical disease and the potential to spread AIV by viral shedding is highly variable between bird species and may depend on the AIV HA subtype [8]. Domestic poultry species such as chicken and turkey are susceptible to only a limited range of circulating influenza virus subtypes [9]. Infections with low pathogenic (LP) AIV in commercial poultry may result in respiratory disease, drop in egg production and increased mortality, whereas natural host species normally show no clinical signs after infection [10]. During the last decade, AIV subtypes H5, H6, H7 and H9 played the major role in influenza outbreaks in poultry in Eurasia [11], [12]. The H5 and H7 LPAIV, once introduced into poultry, may mutate to highly pathogenic (HP) AIV, which have significant zoonotic potential [13], [14]. H9N2 AIV have become widespread in Eurasia since the mid-1990's. Viruses of endemic H9N2 sublineages circulating in poultry populations in Asia occasionally transmit to humans and mammals [15]–[17]. The receptor binding specificity of these H9N2 AIV changed from preferential binding to avian-like α2,3-linked sialic acid (Siaα2,3) receptors to preferential recognition of human-like α2,6-linked sialic acids (Siaα2,6) [18], [19]. Several poultry species, such as quail, chicken, and turkey, have been considered to potentially act as intermediate host species for AIV, and hence to contribute to the evolution of human-virus-like H9N2 AIV [20]–[22]. Susceptibility to AIV can be determined at least in part by sialic acid receptor profiles at the primary site of influenza virus infection. Epithelial cells from the respiratory tract of chicken, turkey, duck and pigeon have been shown to possess both Siaα2,3 and Siaα2,6 receptors in different ratios [23], [24], and viruses circulating in different avian species have been shown to differ in their fine receptor-binding specificity (for a review, see ref. [25]).

Our objectives were to investigate the possible role of different avian species in the process of AIV evolution, and to compare the potential adaptation and increase in virulence between different AIV subtypes. To address this objective, we serially passaged LPAIV of subtypes H9N2, H7N7 and H6N8 in tracheal organ cultures (TOC) and primary embryo fibroblasts (EF) of chicken (*Gallus gallus domesticus*), turkey (*Meleagris gallopavo* f. *domestica*), Pekin duck (*Anas platyrhynchos domestica*) and homing pigeon (*Columba livia domestica*). The results demonstrated that susceptibility to AIV varied significantly between cell cultures of different bird species tested, with respiratory cells of turkey being highly susceptible and those of pigeon showing the lowest LPAIV replication rates. Adaptation of LPAIV to TOC and EF of different bird species led to changes in viral growth kinetics, induction of cell death and the development of influenza virus genome mutations. These were found most prominently in the protease cleavage and receptor-binding site (RBS) regions of the hemagglutinin.

## Results

### LPAIV replication kinetics and ciliostasis in tracheal organ cultures (TOC)

**Figure 1:**
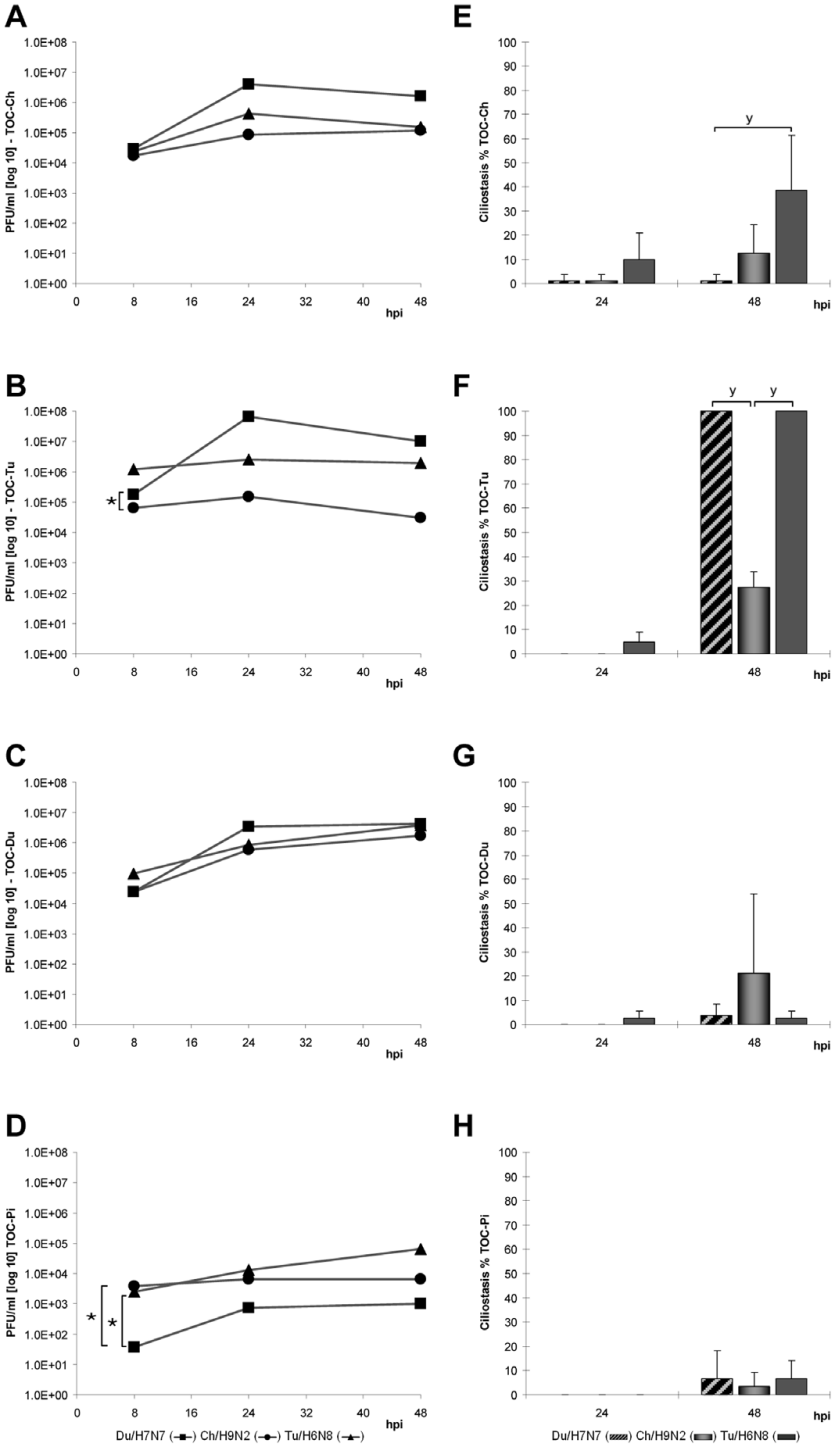
Virus infection in TOC of different avian species. Replication kinetics (A–D) and induction of ciliostasis (E–H) by LPAIV Du/H7N7, Ch/H9N2 and Tu/H6N8 during the first passage. For each time point and virus, groups of four individual TOC-Ch (A,E), TOC-Tu (B,F), TOC-Du (C,G) and TOC-Pi (D,H) were inoculated with 10^4^ PFU of virus in 100 µl PBS+0.2% BSA. Supernatants were harvested at 8, 24 and 48 hours post inoculation, pooled for each TOC group and titrated. Ciliostasis was assessed in percent loss of ciliary activity for each TOC using an inverted microscope (n = 4); error bars indicate the standard deviation. “★” indicate statistically significant differences between virus titer curves (p<0.05; Randomized Complete Block ANOVA, Tukey HSD) and “**y**” between ciliostasis of TOC (p<0.05; Kruskal Wallis test, Wilcoxon Rank sum test).

All viruses replicated in the first passage in TOC of each bird species without addition of exogenous proteases (Fig. 1). The replication rates of the different LPAIV varied between TOC of the four bird species (Fig. 1 A–D). Inoculation of TOC of homing pigeon (TOC-Pi) resulted in the lowest viral titers for all tested AIV strains. In contrast, efficient virus replication of all subtypes was observed already at eight hours post inoculation (hpi) in TOC of turkey (TOC-Tu) and TOC of chicken (TOC-Ch). They reached peak titers at 24 hpi. TOC of Pekin duck (TOC-Du) and homing pigeon showed maximum viral titers later at 48 hpi for all tested LPAIV. Du/H7N7 reached the highest titers compared to other viruses in TOC-Ch, TOC-Tu and TOC-Du, but interestingly it replicated poorly in TOC-Pi. Comparing virus growth kinetics in different avian species, Tu/H6N8 showed the most efficient replication rates in TOC-Tu, whereas Ch/H9N2 replicated to the highest titers in TOC-Du.

**Figure 2:**
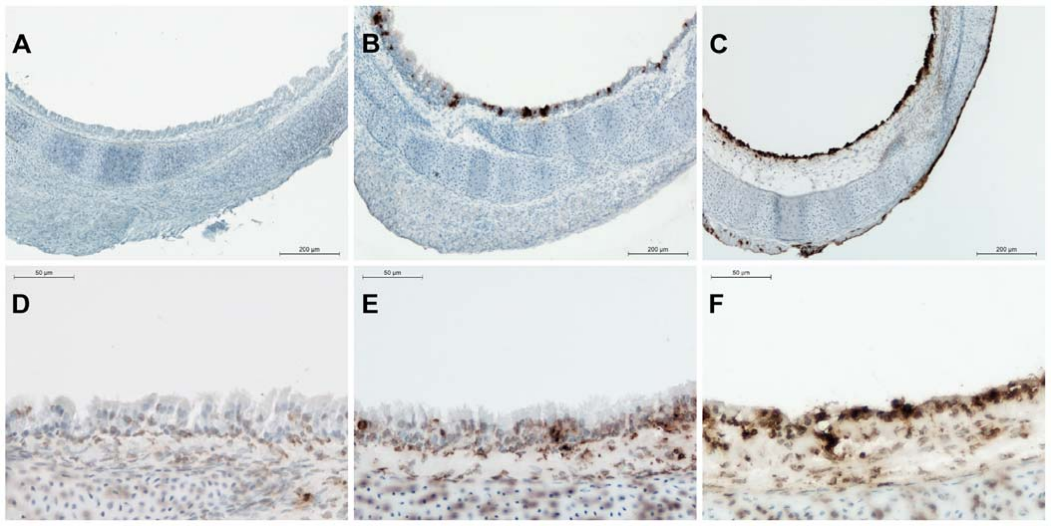
Detection of viral antigen (A,B,C) and induction of apoptosis (E,F,G) in TOC-Ch after infection with Ch/H9N2. TOC-Ch were infected with 10^4^ PFU of Ch/H9N2 in the third consecutive passage or with PBS as non-infected control. Cryosections of TOC were used for immunohistochemical staining for viral nucleoprotein (A,B,C). (A) Non-infected TOC-Ch at 8 hpi, (B) Ch/H9N2-infected TOC-Ch at 8 hpi, (C) Ch/H9N2-infected TOC-Ch at 24 hpi. Non-infected TOC-Ch were negative for viral antigen staining at 24 hpi. *In situ* TUNEL staining for apoptosis (D,E,F). (D) Non-infected TOC-Ch at 24 hpi (no difference to non-infected TOC-Ch at 8 hpi), (E) Ch/H9N2-infected TOC-Ch at 8 hpi, (F) Ch/H9N2-infected TOC-Ch at 24 hpi. Brown staining indicates the presence of viral antigen (A,B,C) or apoptotic cells (D,E,F).

The replication of AIV in the respiratory epithelium of TOC was confirmed by immunohistochemistry (IHC) against viral nucleoprotein. The third consecutive passage of each virus in TOC-Ch was investigated and IHC-staining was seen in the respiratory epithelium of virus-infected TOC at 8, 24 and 48 hpi, which increased over time (Fig. 2 A–C). At 8 hpi, all viruses showed mild positive staining in respiratory epithelial cells, while at 24 hpi, nearly all epithelial cells of the respiratory tract were positive for influenza A NP-staining, as well as some cells of the TOC-surrounding connective tissue.

The different LPAIV induced ciliostasis in the respiratory epithelium at different time points (Fig. 1 E–H). Differences were observed between virus subtypes as well as bird species in the onset time and magnitude of induced ciliostasis. In the first passage, complete ciliostasis was seen in TOC-Tu at 48 hpi with Du/H7N7 and Tu/H6N8, which showed significantly more ciliostasis compared to TOC of other avian species (p<0.05). The most significant ciliostasis was seen in TOC-Ch after Tu/H6N8 infection. By contrast, in TOC-Du only Ch/H9N2 showed some ciliostasis at 48 hpi. Overall, TOC-Pi were the least sensitive to AIV-induced ciliostasis up to 48 hpi. Mock-inoculated TOC did not develop any ciliostasis (data not shown).

Degeneration of the respiratory epithelium was confirmed by histology and IHC staining for apoptotic cells in TOC-Ch (Fig. 2 D–F). All virus-infected TOC-Ch showed loss of cilia, cell degeneration as well as induction of apoptosis (score 2–3) in the respiratory epithelium beginning at 24 hpi (Fig. 2 F). The histopathologic picture was even more severe at 48 hpi. The distribution of viral antigen in the infected tissues correlated well with the induction of apoptosis at all tested time points (Fig. 2). Non-infected TOC-Ch only showed single apoptotic cells in the respiratory epithelium (score 1) and were negative for viral antigen by IHC at the investigated time points.

### LPAIV replication kinetics and cytopathic effects in embryo fibroblasts (EF)

In contrast to TOC, which contain endogenous virus-activating protease(s), LPAIV could only replicate efficiently in EF when trypsin was added to the culture medium. In this case, the replication rates after inoculation of primary EF cultures of chicken, turkey, Pekin duck and homing pigeon in general agreed with the results obtained using corresponding TOC.

In the first passage, inoculation of EF of homing pigeon (PEF) resulted in the lowest viral titers, whereas in EF of turkey (TEF) all viruses propagated to the highest titers compared to EF of other avian species (Table 1). Inoculation of EF of chicken, turkey and Pekin duck resulted in the same growth pattern of different virus strains as seen in TOC. In the first passage Du/H7N7 replicated to the highest and Ch/H9N2 to the lowest titers except in Pekin duck, where Tu/H6N8 showed lowest titers. Cytophatic effects (CPE) were observed in virus-infected EF of chicken, turkey and Pekin duck origin, whereas no CPE was detected in PEF. No differences in the intensity of CPE were observed between virus strains at the different time points in any EF of the different avian species. However, CPE were seen clearly in CEF and TEF at 24 hpi in contrast to DEF, where CPE were observed later at 48 hpi.

**Table 1:** Mean titers of viruses (log _10_ PFU/ml) in EF cultures 48 hpi.

EF origin	Passage	Du/H7N7	Ch/H9N2	Tu/H6N8
Chicken	1	6.80	5.08	6.46
	3	7.18	7.32	6.61
Turkey	1	7.38	5.78	6.61
	3	6.98	7.18	6.93
Pekin duck	1	5.84	5.45	4.99
	3	6.72	6.94	5.95
Homing pigeon	1	3.26	3.26	3.68
	3	3.68	5.68	4.38

### Virus adaptation to TOC and EF of different avian species

**Figure 3:**
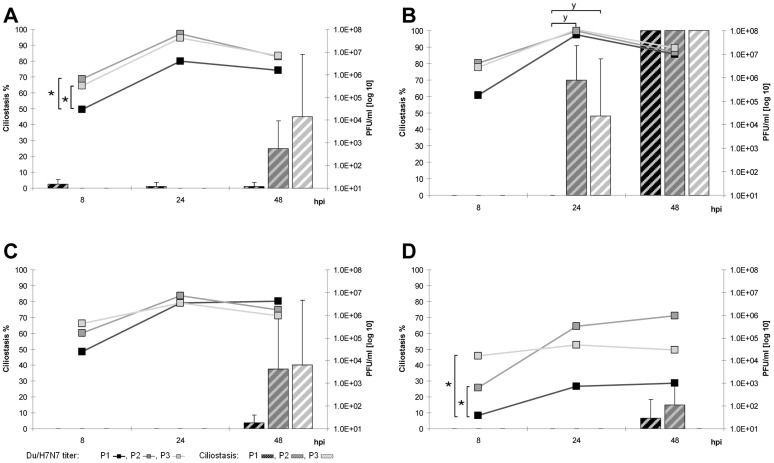
Adaptation of Du/H7N7 to avian TOC. Replication kinetics and induction of ciliostasis over three passages (P1–P3) in TOC-Ch (A), TOC-Tu (B), TOC-Du (C) and TOC-Pi (D). Mean viral titers (PFU/ml) are presented as curves, the percentage of ciliostasis is shown as columns; error bars indicate the standard deviation. “★” indicate statistically significant differences between virus titers (p<0.05; Randomized Complete Block ANOVA, Tukey HSD) and “**y**” between ciliostasis of TOC (p<0.05; Kruskal Wallis test, Wilcoxon Rank sum test).

**Figure 4:**
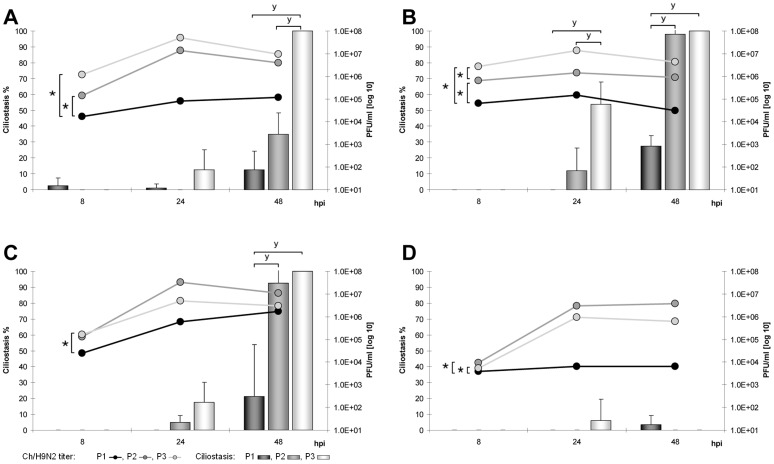
Adaptation of Ch/H9N2 to avian TOC. Replication kinetics and induction of ciliostasis over three passages (P1–P3) in TOC-Ch (A), TOC-Tu (B), TOC-Du (C) and TOC-Pi (D). Mean viral titers (PFU/ml) are presented as curves, the percentage of ciliostasis is shown as columns; error bars indicate the standard deviation. “★” indicate statistically significant differences between virus titers (p<0.05; Randomized Complete Block ANOVA, Tukey HSD) and “**y**” between ciliostasis of TOC (p<0.05; Kruskal Wallis test, Wilcoxon Rank sum test).

**Figure 5:**
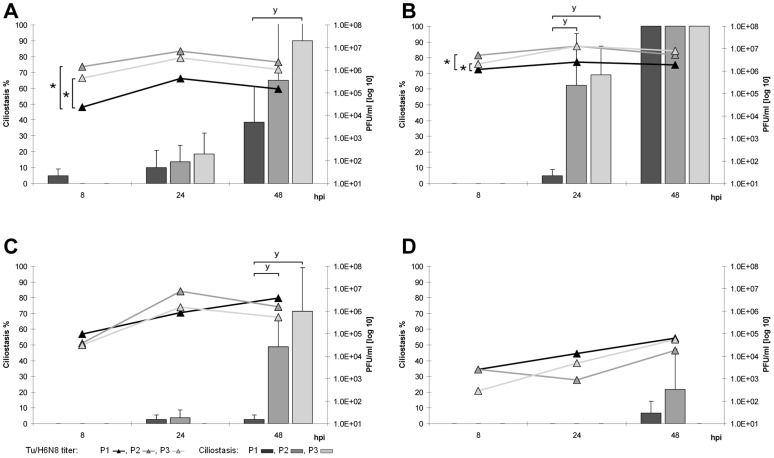
Adaptation of Tu/H6N8 to avian TOC. Replication kinetics and induction of ciliostasis over three passages (P1–P3) in TOC-Ch (A), TOC-Tu (B), TOC-Du (C) and TOC-Pi (D). Mean viral titers (PFU/ml) are presented as curves, the percentage of ciliostasis is shown as columns; error bars indicate the standard deviation. “★” indicate statistically significant differences between virus titers (p<0.05; Randomized Complete Block ANOVA, Tukey HSD) and “**y**” between ciliostasis of TOC (p<0.05; Kruskal Wallis test, Wilcoxon Rank sum test).

To assess the adaptation potential of the LPAIV to different avian hosts *in vitro*, three consecutive passages of each virus were performed in TOC and EF cultures of chicken, turkey, Pekin duck and homing pigeon. Growth characteristics of Du/H7N7, Ch/H9N2 and Tu/H6N8 changed during these passages and revealed avian species-specific differences (Fig. 3, 4, 5).

Du/H7N7 showed increased replication rates in TOC of all tested species already in the second passage and replicated over the three passages most effectively in TOC-Tu and TOC-Ch compared to other species (Fig. 3 A–D). In TOC-Pi, virus titers were strongly increased at later passages, but did not reach the titers observed in TOC of other species. Du/H7N7-induced ciliostasis increased during the three passages in TOC of all species with the exception of TOC-Pi. Du/H7N7-infected TOC-Tu showed significantly enhanced ciliostasis in the second passage as early as 24 hpi compared to other species (p<0.05).

In the third passage, replication rates of Ch/H9N2 increased significantly (p<0.05) compared to the first passage in TOC of all tested bird species but with species-specific differences (Fig. 4 A–D). Ch/H9N2 replicated to already high titers in the second passage as early as eight hpi in TOC-Tu, TOC-Ch and TOC-Du in contrast to TOC-Pi. In the third passage, growth curves of Ch/H9N2 reached their peak already at 24 hpi in TOC of all investigated avian species, while it took 48 hpi in the first passage in TOC of all tested birds except turkey. In the second and third passage, ciliostasis was enhanced only in TOC-Tu, TOC-Ch and TOC-Du but not in TOC-Pi with the highest percentages seen for turkey.

Serial passaging of Tu/H6N8 showed statistically significantly increased titers in TOC-Tu and TOC-Ch in the second and third passage (Fig. 5 A–D). In TOC-Pi, the replication rates of Tu/H6N8 remained on a low plateau during all passages. Tu/H6N8-induced ciliostasis most significantly increased in TOC-Tu compared to other species. Tu/H6N8-induced ciliostasis in TOC-Ch was observed as early as 24 hpi over all three passages, in contrast to the other two LPAIV strains.

TOC-Pi were additionally analyzed for ciliostasis up to seven days pi. All tested LPAIV failed to induce ciliostasis in TOC of homing pigeon at any of the later time points of the three passages (data not shown).

Statistically significant increased replication rates pi (p<0.05) were seen in EF of all avian species for Ch/H9N2, but for Tu/H6N8 and Du/H7N7 only in DEF in the third passage (Table 1). Only Ch/H9N2 induced a slightly enhanced CPE in EF of chicken, turkey and Pekin duck, whereas it was strongly enhanced in EF of pigeon at 48 hpi in the third passage (data not shown).

### Mutations in the viral genes after three passages

HA, PB2 and NS genes of third passage viruses were sequenced and compared with the original virus sequences. We focused mainly on these genes because they are known to play a critical role in the virus host range and interspecies transmission [7]. Amino acid (aa) substitutions and silent mutations in the HA, PB2 and NS segments were found in TOC- and EF-adapted viruses. Mutations of the HA were seen in all tested LPAIV (Table 2). For some viruses, HA mutations were found at the cleavage site region of the HA0 precursor protein (Ch/H9N2 substitution A325V: PARSSR*GLF>PVRSSR*GLF; Tu/H6N8 substitution E327V: PQAETR*GLF>PQAVTR*GLF). The majority of HA mutations were located either in the receptor binding pocket (amino acid positions 137 and 226) or in its vicinity (position 158). Plaque purification and subsequent sequencing of TOC-Tu-adapted Ch/H9N2 virus confirmed presence of all three mutations in eight out of ten randomly picked plaques. Silent mutations occurred in the HA of PEF-adapted Tu/H6N8 (nt C985T, nt G1632A).

**Table 2:** Amino acid substitutions in the hemagglutinin after three passages in TOC and EF.

Virus	Species	Culture^1^	Substitution^2^	Position^3^	Location^4^
Ch/H9N2	Turkey	TOC	N→S*	158	HA head
			Q→I	226	Receptor binding pocket
			I→V	267	Head-stalk interface of HA1
Ch/H9N2	Turkey	EF	A→V	325	Cleavage site
Ch/H9N2	Pekin duck	TOC	N→S*	158	HA head
Ch/H9N2	Pigeon	TOC	A→V	325	Cleavage site
Tu/H6N8	Chicken	TOC	N→Y	244	HA1-HA1 interface in trimer
Tu/H6N8	Pekin duck	TOC	N→Y	244	HA1-HA1 interface in trimer
Tu/H6N8	Turkey	EF	E→V	327	Cleavage site
Du/H7N7	Turkey	EF	G→D	137	Receptor binding pocket
Du/H7N7	Chicken	EF	G→D	137	Receptor binding pocket

1 TOC: Tracheal organ culture; EF: Embryo fibroblast culture.

2 Compared to wild-type virus.

3 H3 numbering system [62].

4 See Figure 6.

* Mutation destroys potential glycosylation site.

The PB2 of the Ch/H9N2 acquired two amino acid substitutions (E472K and L571I) after three passages in TOC and EF of all tested bird species, one additional mutation was found after passaging of the virus in PEF (Table 3). The polymorphisms of amino acids at certain positions in the consensus sequence of the sequenced virus population ranged semiquantitatively from 30% (TOC-Tu, TOC-Ch, CEF, PEF) to 50% (TOC-Du, TEF, DEF) and 90% (TOC-Pi). Silent mutations of the PB2 of Ch/H9N2 were found in TOC-Pi and TEF (nt A711G) as well as in PEF (nt G1851A). The PB2 of Tu/H6N8 showed a single silent mutation in TOC-Pi (nt C1878A).

**Table 3:** Amino acid substitutions in PB2 and NS after three passages.

Virus	Species	Culture^1^	PB2	NS1
Ch/H9N2	Homing pigeon	TOC	E472K, L571I	V18I
		EF	I382T, E472K, L571I	
	Turkey	TOC	E472K, L571I	
		EF	E472K, L571I	V18I
	Chicken	TOC	E472K, L571I	
		EF	E472K, L571I	
	Pekin duck	TOC	E472K, L571I	
		EF	E472K, L571I	

1 TOC: Tracheal organ culture; EF: Embryo fibroblast culture.

The NS1 protein of Ch/H9N2 showed an identical aa substitution after three consecutive passages in TOC-Pi as well as in TEF (Table 3). Single silent mutations were found in the NS1 sequence of Tu/H6N8 in TOC-Pi, (nt G174A) and PEF (nt G651A) and in the spliced NEP sequence of Ch/H9N2 in TOC-Pi (nt G294A).

The complete viral genome of Ch/H9N2 was sequenced after three passages in TOC of all tested species and compared to the wild-type virus, since it showed the highest mutation rate in the HA, PB2 and NS1 proteins. No predicted aa substitutions were detected in the PB1, PA, NP, NA and M1/M2 proteins after three consecutive passages in TOC of all tested species. Silent mutations were found only in the Ch/H9N2 PA sequence after passaging in TOC-Pi (nt A522G, A1479G, A1680C, A1905G).

Furthermore, the PB1, PA and NA genome segments of Du/H7N7 and Tu/H6N8 LPAIV were analysed for mutations after passaging in TOC-Ch. No mutations were detected in these genome segments.

## Discussion

In order to investigate the adaptation potential of LPAIV of different subtypes to different avian species, we studied an *in vitro* infection of LPAIV isolates Ch/H9N2, Tu/H6N8 and Du/H7N7 in TOC and EF of chicken, turkey, Pekin duck and homing pigeon. TOC mimic the natural local AIV-infection site in the respiratory tract of avian hosts and allow investigations under controlled conditions [26], [27]. TOC are organ cultures with an intact respiratory epithelium consisting of ciliated as well as goblet cells without detectable undifferentiation during culture in either investigated species as indicated by histological investigations (data not shown). TOC-studies are useful to analyze local influenza virus growth characteristics in the presence of innate immune cell mechanisms, but the influence of specific mechanisms and systemic reactions on the infection cannot be studied in an organ-culture.

All tested LPAIV showed clear tropism for the respiratory epithelium of infected TOC, which was confirmed by virus-antigen staining. Infected TOC-Ch showed an increase of viral nucleoprotein staining at later time points. Since reliable quantification of viral-antigen positive cells of the respiratory epithelium was not possible at later time points, correlations to viral titers could not be drawn.

The induction of ciliostasis in TOC upon LPAIV infection allowed speculating on the virulence of LPAIV in the primary target cells of the respiratory epithelium. Ciliostasis is the result of necrosis or apoptosis of ciliated cells of the respiratory epithelium [28]. All tested LPAIV have been shown to be strong inducers of apoptosis upon infection in TOC-Ch. The induction of apoptosis is known to be an important virulence factor of influenza viruses, which is regulated by the influenza virus NS1 protein [29]. In a recent study, H9N2 LPAIV has been shown to replicate and also induce apoptosis in human tracheobronchial epithelial cells [28]

Earlier induction of ciliostasis was seen during the adaptation process of the tested LPAIV in TOC. This observation correlated positively with the increase of viral titers at the designated time points. The assessment of ciliostasis of avian TOC is a widely used tool to analyze the growth behavior and pathogenesis of influenza A viruses as well as other avian viruses, which target cells of the respiratory tract, such as the infectious bronchitis virus (IBV) [30], [31]. Primary EF of different avian species were used as a standard *in vitro* system to characterize influenza viruses and growth behavior [32].

Since all tested species possess different sialic acid receptor profiles in their tracheas [23], [24], we analyzed the susceptibility to different LPAIV strains in TOC, as well as the potential to adapt to the respiratory epithelium of new bird species.

Domestic turkeys possess a high potential to alter and transmit AIV to new host species [20]. They are considered to be susceptible to a wider range of influenza subtypes than chickens, and furthermore develop severe clinical disease after *in vivo* LPAIV and HPAIV infection [33], [34]. Our results confirm that TOC of turkey were the most susceptible for AIV-infection with high replication rates already at 8 hpi accompanied by pronounced ciliostasis.

Chicken TOC were also susceptible to all tested strains, while Du/H7N7 replicated to the highest titers and Tu/H6N8 induced the most severe ciliostasis. All tested LPAIV subtypes also replicated in cells of Pekin duck but with lower rates compared to chicken and turkey.

Pigeons are generally considered to be relatively resistant to influenza virus infection and to play only a minor epidemiological role in influenza virus transmission [35]–[37]. Although surveillance studies showed several incidences of different LPAIV subtypes in pigeons in the field, *in vivo* influenza infection studies with different AIV subtypes mostly failed to induce successful virus replication and disease [8], [38]–[44]. Our studies support this observation by showing that LPAIV-infected TOC-Pi released significantly lower virus titers compared to the other tested bird species and did not reveal significant signs of infection-mediated ciliostasis.

In a comparative study, lectin staining of chicken, duck and turkey revealed the presence of both α2,3 Sias and α2,6 Sias in the respiratory epithelium of tracheas with 90% and 20–90% positive cells respectively, depending on the age and avian species [23]. In contrast, mainly α2,6 Sias were found in pigeon tracheas [24]. Only few cells were positive for α2,3 Sias, which may explain the resistance of pigeons to AIV, which preferably bind to α2,3 Sias.

Furthermore, differences in innate immune reactions may contribute to the difference in species related susceptibility. Recently it was demonstrated that the retinoic acid-inducible gene I (RIG-I) is present in ducks and plays a role in clearing an influenza virus infection [45]. RIG-I triggers the antiviral interferon response in ducks, contributing to the relatively high resistance to AIV infection in this species. In contrast, chickens do not bear RIG-I, which could explain the increased susceptibility to influenza viruses compared to ducks [45]. It may be speculated that cells of the pigeon's respiratory tract may possess also innate immune mechanisms to suppress AIV replication. This aspect needs further investigations.

During interspecies transmission, AIV need to adapt to the new host in order to overcome existing host-range barriers and increase replication. In our study, the increased viral replication rates up to the third passage were likely due to adaptive mutations which allowed more efficient virus replication in the host tissue. Single mutations or a combination of only few amino acid substitutions have been shown to improve virus propagation in new hosts and display an important prerequisite for interspecies transmission [46].

Comparing the investigated virus subtypes, Ch/H9N2 showed the highest mutation rate within the three consecutive passages in TOC and EF cultures. This virus developed HA mutations in TOC of turkey, Pekin duck and homing pigeon as well as in EF culture of turkey, but not in primary cultures of the original host species chicken. These results suggest that the observed mutations allowed the virus to adapt to new host species. Du/H7N7 acquired mutations only after propagation in EF of turkey and Pekin duck. Tu/H6N8 mutated only after passaging in TOC-Ch and TOC-Du.

**Figure 6:**
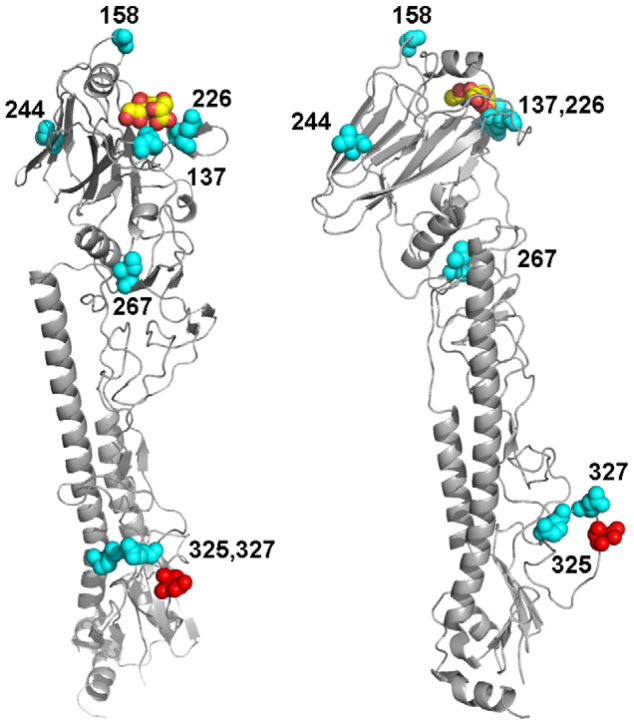
Adaptive mutations in the HA of H9N2, H6N8 and H7N7 shown on the model of H3 HA monomer. The model is based on the crystal structure of non-cleaved HA precursor (1HA0.pdb) [65]. The mutations described in Table 2 are shown in cyan and numbered. Sialic acid in the receptor-binding pocket is represented as space-filled model (carbon and oxygen atoms in yellow and red, respectively). Position of Arg329, which separates HA1 and HA2 in the HA0 precursor, is shown in red. Left and right images display different views of the same model to better illustrate location of mutations.

Mutations of functional HA regions have been shown to be responsible for successful adaptation to new host species [47], [48]. The observed HA cleavage site mutations of Ch/H9N2 and Tu/H6N8 may affect the cleavage efficiency of the HA0 precursor by host cell proteases in the new avian species [49]. These mutations have yet not been described before neither *in vivo* nor *in vitro* and their role in AIV-infection of the different hosts needs to be elucidated further in animal experiments. It may be speculated that the change in cleavage efficacy may also lead to a more vigorous virus replication in the host followed by more severe clinical disease. Substitution N244Y is located at the interface between the HA monomers and could affect a fusion-promotion activity and stability of the molecule [47]. A conservative substitution I267V is located in the region between the globular head and stalk of the HA1. The potential functional significance of this substitution is not clear. Amino acid substitutions Q226I (Ch/H9N2) and G137D (Du/H7N7) involve residues that directly interact with the sialic acid receptor (Fig. 6). In particular, position 226 is known to play an important role in the virus binding preference for either Siaα2,3 or Siaα2,6 [47], [48]. Furthermore, avian H9N2 viruses with Q226L show preferential binding to 2–6-linked receptors [18], [19]. Substitution N158S removes a potential glycosylation site from the top of the HA close to the receptor binding pocket (Fig. 6). Previous studies revealed significant effects of N-linked glycan at this position on receptor-binding properties [50]–[52]. The latter two HA mutations are well described as natural occurring mutations in the field with high relevance for the evolution of human-like H9N2 viruses [53]. We hypothesize that the combination of HA-mutations of the TOC-Tu adapted Ch/H9N2 might have altered the receptor binding preference of the virus from Siaα2,3 towards Siaα2,6, suggesting potential role of turkey in the emergence of viruses with human-virus-like receptor specificity.

Substitution V18I, which is located in the RNA binding domain, may affect binding of different RNA target structures by the NS1 which elicits the NS1 protein in countering the host cell antiviral defence [54].

The PB2 forms together with PB1, PA and NP the influenza virus polymerase complex, which is necessary for virus replication in the host cell [48]. Mutations of the PB2 have been shown to enhance the polymerase activity with increased virulence in a mouse model [55]. The PB2 mutations E472K and L571I may enhance the potential of virus replication in TOC and EF systems of all four avian species although the polymorphisms of amino acids at certain positions may display different selective pressures between cultures for these mutations. Influenza virus genome database analysis indicates that no wild-type viruses with these mutations have been described so far (http://www.ncbi.nlm.nih.gov/genomes/FLU/FLU.html).

No additional mutations were observed in the PB1, PA, NP, NA and M1/M2 proteins of Ch/H9N2 after three consecutive passages in TOC of all four bird species. Also Tu/H6N8 and Du/H7N7 showed no additional mutations in the PB1, PA and NA proteins after adaptation in TOC-Ch. We cannot rule out that mutations in these proteins may have occurred in the adaptation process after further passages in TOC or may have occurred *in vivo* in the different avian species. This needs to be further investigated.

Our study demonstrated different susceptibilities of the analyzed bird species to LPAIV using TOC as an *in vitro* model. The LPAIV strain Ch/H9N2 showed particularly high potential to adapt to new avian species within a low number of serial passages. Mutations of the HA receptor binding site and cleavage site in cells of avian species other than the originated host provide circumstantial evidence that the HA plays a significant role in the AIV host range and transmission between avian species. *In vitro* studies in organ cultures such as TOC are an excellent system to provide the necessary data on possible adaptive mechanisms under controlled conditions. They are a prerequisite for possible further *in vivo* studies and can not be supplemented by *in vivo* studies, which anyway do not allow testing for different viruses in four different species under comparable experimental conditions. Further studies are needed to determine if the adapted viruses show increased potential to infect and replicate in mammalian cells, which would possibly enhance the risk of interspecies transmission.

## Materials and Methods

### Viruses

A/chicken/Saudi Arabia/CP7/1998 (H9N2) (Ch/H9N2), a field isolate from a meat-type chicken flock, was kindly provided by Hans-Christian Philipp from Lohmann Tierzucht (Cuxhaven, Germany). A/turkey/Canada/1963 (H6N8) (Tu/H6N8) was kindly provided by Klaus Peter Behr from AniCon Labor (Hoeltinghausen, Germany). Tu/H6N8 has a 23 amino-acid deletion in the neuraminidase stalk region [56], a genetic marker for influenza virus adaptation to poultry. A/duck/Potsdam/15/1980 (H7N7) (Du/H7N7) was obtained from the Friedrich-Loeffler-Institute, Federal Research Institute for Animal Health (Greifswald-Insel Riems, Germany). The hemagglutinin cleavage site sequence of Du/H7N7 (PEIPKGR*GLF) classifies the virus as low-pathogenic (LP). All viruses were propagated in 10-day-old embryonated, specific-pathogen-free chicken eggs (VALO-SPF, Lohmann Tierzucht, Cuxhaven, Germany).

### Virus titration

Infectious virus titers were determined using plaque assay in Madin-Darby canine kidney (MDCK; obtained from the Institute for Medical Virology, Justus-Liebig-University Giessen, Germany [57]) cells under Avicel overlay medium as previously described [58]. MDCK cells were seeded in 6-well plates (Sarstedt, Nuembrecht, Germany). Confluent MDCK monolayers were incubated in duplicate wells with 400 µl of serial 10-fold dilutions of viruses in PBS-BSA (phosphate buffered saline [PBS] containing 0.2% bovine serum albumin [BSA; PAA Laboratories, Pasching, Austria]) for 1 h. After washing with PBS, the cells were covered with low-viscosity overlay medium consisting of DMEM (Biochrom, Berlin, Germany), 1.5% Avicel RC-581 (FMC Biopolymer, Ratingen, Germany), 0.2% BSA and 1 µg/ml acetylated trypsin (Sigma-Aldrich, Taufkirchen, Germany). After an incubation period of 48 h at 37.5°C and 5% CO_2_, cells were fixed with 4% paraformaldehyde (Sigma-Aldrich) in PBS and stained with 1% crystal violet (Chroma, Münster, Germany). Plaques were counted, and infectious titers were calculated from the mean of duplicate wells as plaque-forming units (PFU) per ml using the formula:
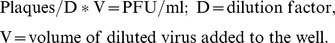
A standard hemagglutination assay with 1% chicken red blood cells was used to determine the hemagglutinating titers of the virus stocks [59].

### Organ and cell cultures

Tracheal organ cultures were prepared from embryonated eggs of chicken (VALO-SPF, Lohmann Tierzucht, Cuxhaven, Germany) at incubation day 20 (TOC-Ch), turkey (Moorgut Kartzfehn, Boesel, Germany) at incubation day 26 (TOC-Tu), Pekin duck (Duck-Tec, Belzig, Germany) at incubation day 25 (TOC-Du), and homing pigeon (local non-commercial breeder) at incubation day 17 (TOC-Pi) [26], [27]. All parental flocks were tested negative for AIV-antibodies of subtypes H5, H7, H6 and H9 in the hemagglutination inhibition (HAI) test.

Briefly, embryos were sacrificed, and the tracheae were removed under sterile conditions. Each trachea was cut manually into approximately 0.8 mm thick rings using a microtome blade. Individual rings were transferred to 5 ml tubes (Sarstedt, Nuembrecht, Germany) with 0.8 ml prewarmed Medium 199 with Hanks' salts (Biochrom) including 1% penicillin/streptomycin (P/S; 10.000 U/ml, 10.000 µg/ml, Biochrom). TOC were cultured at 37.5°C in a rotating shaker (Reax 2, Heidolph, Schwabach, Germany) at lowest rotation speed.

After 24 h, the ciliary activity of the respiratory epithelium of each TOC was assessed using an inverted microscope. Only rings with 100% ciliary activity were used for the experiments. TOC were infected 5 days after preparation to avoid negative effects due to early inflammatory responses of the tissue [60]. Overall, the viability of non-infected TOC was stable at least for four weeks without any loss of cilia activity. Histological investigations of selected TOC from different avian species confirmed the perpetuation of the cell structure of the respiratory epithelium and underlying tissue over the infection experiments. Preliminary studies also confirm the expression of Siaα2,3 and Siaα2,6 during infection experiments indicating the stability of the *ex vivo* cultures [30].

Primary embryo fibroblast (EF) cultures were prepared according to standard protocols [61] from embryonated eggs of chicken (CEF), turkey (TEF), Pekin duck (DEF) and homing pigeon (PEF) at day 10, 13, 14 and 9 of incubation, respectively. EF of all four species were seeded in 24 well plates (Sarstedt, Nuembrecht, Germany) and maintained in 1∶1 Mc Coy's 5A modified Medium+L-15 Leibovitz Medium (Biochrom) with 10% fetal bovine serum (FBS) (Biochrom), 1% L-glutamine (200 mM, Biochrom) and 1% P/S at 37.5°C and 5% CO_2_.

MDCK cells were cultured in Dulbecco's MEM (Biochrom) with 10% FBS, 1% L-Glutamine and 1% P/S at 37.5°C and 5% CO_2_.

### Ethics statement

A study approval from an ethics committee was not required, since working with avian embryos is currently not regulated by legislation as animal experimentation in Germany (http://www.bmelv.de/SharedDocs/Rechtsgrundlagen/T/Tierschutzgesetz.html), as confirmed by the animal welfare official of the University of Veterinary Medicine Hannover. Protocols for working with embryonated eggs were in accordance with the European Union Legislation (http://eur-lex.europa.eu/LexUriServ/LexUriServ.do?uri=CELEX:32005L0094:EN:NOT). Embryos used for tracheal organ culture or embryonic fibroblast preparation did not undergo any procedures prior to humane sacrifice by decapitation. Virus propagation was carried out in embryonated chicken eggs inoculated at incubation day 10, maintained for three days and subsequently chilled at 4°C for 24 h.

Section § 8a paragraph 1 a. 2 says:

“Any person intending to conduct experiments on vertebrates for which no authorization is required or on cephalopods or decapods shall notify the planned experiment to the competent authority at least two weeks before the experiment begins. This time limit need not be observed in emergencies where the experiment must be carried out immediately. In this case notification shall be sent immediately afterwards. The time limit referred to in the first sentence may be extended by the competent authority, if required, to up to four weeks.

(2) The notification shall indicate:

1. the purpose of the planned experiment;
2. the species and, in the case of vertebrates, also the number of animals to be used for the planned experiment;
3. the type of animal experiments planned and the procedures to be used, including anaesthetization;
4. the place, beginning and likely duration of the planned experiment;
5. the name, address and expertise of the head of the experiment in charge, of his deputy and of the person performing the experiment as well as of the eligible persons for after-treatment;
6. in the case of planned experiments pursuant to Article 8, paragraph 7 (1) the legal basis for exemption from authorization.”

http://www.animallaw.info/nonus/statutes/stdeawa1998.htm

### Virus sequencing

The viral genome segments encoding hemagglutinin (HA), polymerase basic protein 2 (PB2) and nonstructural protein (NS) of all three tested LPAIV were analyzed for mutations after three consecutive passages in EF and TOC of all four bird species. Based on the obtained results, Ch/H9N2 was selected as the most adaptive virus and all other AIV genome segments, namely polymerase basic protein 1 (PB1), polymerase acidic protein (PA), nucleoprotein (NP), neuraminidase (NA) and matrix protein (M1/M2) were sequenced after the third passage in TOC of either of the different species. Additionally, the PB1, PA and NA genome segments of Tu/H6N8 and Du/H7N7 were sequenced after three consecutive passages in TOC-Ch. The viral sequences obtained after the third passage were compared to those of the virus stocks to detect nucleotide and amino acid changes. The H3 numbering system [62] was used for amino acid substitutions in the HA.

Briefly, total RNA was extracted from EF and TOC culture supernatants and allantoic fluids of the virus stocks by QIAamp Viral RNA Mini Kit (Qiagen, Hilden, Germany). RT-PCR amplification was carried out with universal primers described by Hoffmann et al. [63] and Li et al. [64] using the SuperScript One-Step RT-PCR System with Platinum Taq High Fidelity polymerase (Invitrogen, Carlsbad, CA). Following gel electrophoresis, specific DNA-bands were selected and purified with the Silica Bead DNA Gel Extraction Kit (Fermentas, St. Leon-Rot, Germany). Direct sequencing of the purified products was done by Eurofins MWG Operon (Ebersberg, Germany).

Mutations observed in the HA and PB2 of Ch/H9N2 were confirmed also in plaque purified virus preparations of the supernatant of the third passage in TOC-Tu. Briefly, after incubation of MDCK cells with 50 PFU virus for 1 h, cells were overlayed with DMEM (Biochrom) supplemented with 0.9% Biozym Plaque Agarose (Biozym Scientific, Hessisch Oldendorf, Germany), 0.2% BSA and 1 µg/ml acetylated trypsin. Cells were incubated at 37.5°C and 5% CO2 for 72 h. Ten individual plaques were picked, each propagated in MDCK cell monolayers for 24 h, and subsequently investigated for mutations in the HA and PB2 as described above.

### Virus infection in cell and organ cultures

Three consecutive passages of LPAIV Ch/H9N2, Tu/H6N8 and Du/H7N7 were made under identical conditions in TOC and EF cultures of chicken, turkey, Pekin duck and homing pigeon. Multicyclic replication kinetics was studied by inoculating EF cultures with 0.01 PFU of the virus per cell; TOC were inoculated with 10^4^ PFU of the virus per individual culture using PBS-BSA as dilution medium. Virus-negative controls were incubated with PBS-BSA instead of the virus. After 1 h of incubation, TOC and EF were washed with PBS and subsequently cultured in 1 ml basal maintenance media used for cultivation of corresponding cultures including 0.2% BSA. 1 µg/ml acetylated trypsin was added to EF cultures. In the first passage, TOC and EF cultures were inoculated with virus stocks from embryonated chicken eggs. In the consecutive passages, cultures were inoculated with titrated culture supernatants, which had been collected in the previous passage 24 h postinfection (hpi). At eight, 24 and 48 hpi, supernatants of triplicate EF cultures and four randomly selected TOC were collected and pooled for each virus. Each virus pool was titrated in a plaque assay as described above. Cytopathic effects (CPE) were assessed in EF monolayers (n = 3). TOC were analyzed under the microscope for ciliostasis at the indicated time points for percentage of remaining ciliary activity as described previously [27].

### Immunohistochemical detection of viral antigen

Immunohistochemistry was performed to detect influenza virus nucleoprotein (NP) antigen in TOC. Briefly, TOC cryosections (5 µm) were fixed in ice-cold acetone and blocked against endogenous peroxidase with 0.3% hydrogen peroxide in methanol. Normal horse serum and the Avidin/Biotin Blocking kit (Vector Laboratories, Burlingame, CA) were used to block nonspecific staining. Sections were incubated with mouse anti influenza A nucleoprotein antibody (AbD Serotec, Duesseldorf, Germany) diluted 1∶1000 in PBS followed by the Vectastain Elite ABC kit (Mouse IgG; Vector Laboratories) according to the manufacturer's instructions. Peroxidase activity was developed using the DAB Peroxidase Substrate kit (Vector Laboratories). Sections were counterstained with hematoxylin and mounted with Aquatex (Merck, Darmstadt, Germany).

### Detection of apoptotic cells

Detection of apoptotic cells was performed in TOC cryocections with the TUNEL (terminal transferase-mediated d-UTP nick-end labelling) assay using the *in situ* cell death detection kit POD (Roche Applied Sciences, Mannheim, Germany) according to manufacturer's instructions. Peroxidase activity was developed using the DAB Peroxidase Substrate Kit (Vector Laboratories) according to manufacturer's instructions. TOC sections were counterstained with hematoxylin and mounted with Aquatex. The presence of apoptotic cells was evaluated microscopically in the respiratory epithelium of Ch/H9N2, Tu/H6N8 and Du/H7N7 infected TOC-Ch at 8, 24 and 48 hpi and scored semiquantitatively (1 = 0–5 pos. cells; 2 = 6–50 pos. cells; 3 = >50 pos. cells).

### Statistical analysis

Statistically significant differences between viral titers were evaluated with the Randomized Complete Block Analysis of Variance (ANOVA) and Tukey HSD using Statistix 9.0 (Analytical Software, Tallahassee, FL). Randomized Complete Block ANOVA allows the comparison of different growth curves using the data of multiple timepoints. Significant differences in induction of ciliostasis were assessed with the Kruskal-Wallis test and Wilcoxon Rank sum test (Statistix 9.0). Differences were considered significant at a *P* value of <0.05.

### Nucleotide sequence accession numbers

The nucleotide sequences of the LPAIV obtained in this study can be found under GenBank accession numbers CY081259, CY081261, CY081263, CY081264, CY120060, CY081266, CY120061, CY081268 (Ch/H9N2); CY081270, CY081272, CY081273, CY081275, CY081277, CY081279 (Du/H7N7); and CY081280, CY081282, CY081284, CY081286, CY081288, CY081290 (Tu/H6N8).
